# Electrospinning Fabrication of Poly(vinyl alcohol)/*Coptis chinensis* Extract Nanofibers for Antimicrobial Exploits

**DOI:** 10.3390/nano8090734

**Published:** 2018-09-17

**Authors:** Seong Baek Yang, Eun Hee Kim, Seung Hee Kim, Young Hun Kim, Weontae Oh, Jin-Tae Lee, Young-Ah Jang, Yeasmin Sabina, Byung Chul Ji, Jeong Hyun Yeum

**Affiliations:** 1 Department of Advanced Organic Materials Science and Engineering, Kyungpook National University, Daegu 41566, Korea; ysb@knu.ac.kr (S.B.Y.); yeasminsabina44@knu.ac.kr (Y.S.); bcji@knu.ac.kr (B.C.J.); 2 Gooworl Co., Ltd., Daegu 41422, Korea; coffee7110@naver.com; 3 Korea Research Institute for Fashion Industry, Daegu 41028, Korea; kimsh@krifi.re.kr; 4 Skin Science R&D Center, Gennolab Co., Ltd., Gyeongsan, Gyeongbuk 38541, Korea; 5 Division of Advanced Materials Engineering, Dong-Eui University, Busan 47340, Korea; wtoh2005@deu.ac.kr; 6 Department of Cosmeceutical Science, Daegu Haany University, Gyeongsan, Gyeongbuk 38610, Korea; jtlee@dhu.ac.kr (J.-T.L.); yaviol@nate.com (Y.-A.J.)

**Keywords:** Poly(vinyl alcohol), *Coptis chinensis*, extract, antibacterial, antifungal

## Abstract

*Coptis chinensis* (CC) is used in conventional Chinese medicine. The main active components of CC are isoquinoline alkaloids, including berberine, coptisine, palmatine, and magnoflorine; all these are known to have several pharmacological properties. Poly(vinyl alcohol) (PVA) is a well-known synthetic biocompatible polymer suitable for a range of pharmaceutical uses; it can be used as a matrix for the incorporation of functional materials and has a wide range of applications in the cosmetics, food, pharmaceutical, and packaging industries. In this study, PVA-based electrospun nanofibers containing CC extract were successfully fabricated. Furthermore, the effects of different CC extract contents on the morphologies, and antimicrobial and antifungal properties of PVA/CC extract nanofibers were investigated. Morphological changes were observed using different molecular weights of PVA. For characterization, field-emission scanning electron microscopy, thermogravimetric analysis, and Fourier transform infrared analysis were performed. The effectiveness of these nanofibers has been demonstrated by evaluating the thermal stability against *Staphylococcus aureus*, antimicrobial activity against *Staphylococcus aureus* and *Staphylococcus epidermidis*, and the antifungal activity against the fungi *Aureobasidium pullulans* and *Penicillium pinophilum*. The PVA/CC extract nanofibers were found to have excellent antibacterial and antifungal activity and thermal stability; hence, their use in medicinal sectors is highly recommended.

## 1. Introduction

**Figure 1:**
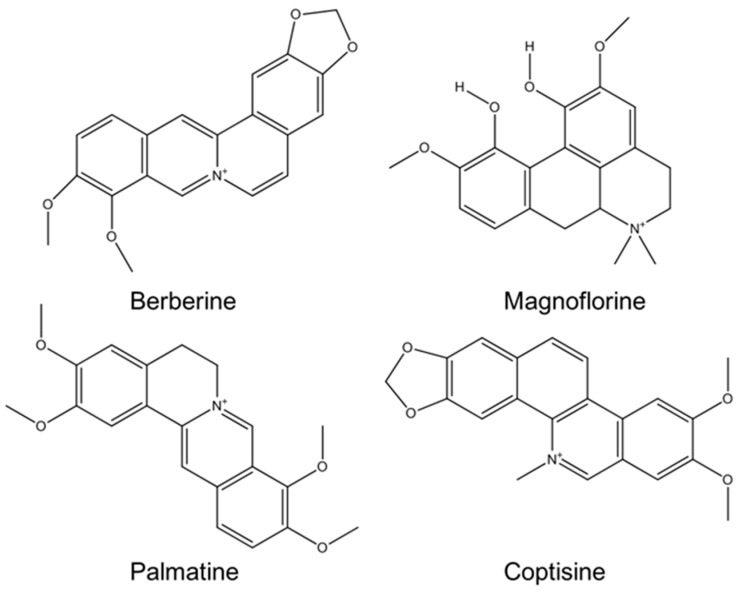
The chemical structures of the major components of the *Coptis chinensis* (CC) extract.

*Coptis chinensis* (CC) is a medicinal plant generally found in western China; for example, in the Sichuan and Shanxi provinces. The root of this plant has been widely used as a traditional Chinese drug for treating diarrhea, fever, and eczema [1]. It comprises a range of alkaloids, including berberine, palmatine, jateorrhizine, epiberberine, and coptisine, which are regarded as its active components [2]. Berberine, a key component of the CC, has been discovered to have a synergistic effect against methicillin-resistant *Staphylococcus aureus* when used with azithromycin and levofloxacin [3]. Palmatine is a very close structural equivalent of berberine, and occurs in members of all plant families containing berberine, but in extremely low amounts. It has been examined for its possible use in the cure of jaundice, dysentery, hypertension, liver-related diseases, and inflammation, and it has been recommended that palmatine could potentially be used for curing flaviviral diseases [4]. Magnoflorine, another component of CC, has demonstrated a varied range of beneficial biological activities; for example, antioxidation, antidiabetic, anti-inflammation, antifungal, antitumor, and antiviral activities [5]. Coptisine is also an alkaloid found in CC. It is well known for producing a bitter taste; it is usually used in Chinese herbal medicine, together with a related compound, berberine, for treating digestive sicknesses caused by bacterial infections [6]. The chemical structure of the major components of the CC is shown in Figure 1.

CC extract can be extracted in different ways. Teng and his group used aqueous ethanol with different proportions of ethanol and water, ranging from 0 to 95% *v*/*v* to extract all alkaloid compounds from CC [7]. In another report, Liu et al. showed an extraction method using supercritical fluid to extract berberine from rhizome of CC and the recovery of berberine was also compared with different modifiers including methanol and 95% *v*/*v* ethanol with and without surfactant Tween 80, and 1,2-propanediol [8]. Besides, accelerated solvent extraction method along with ultra-performance liquid chromatography analysis has been established for the identification and quantification of main alkaloids in CC extracts [9].

**Figure 2:**
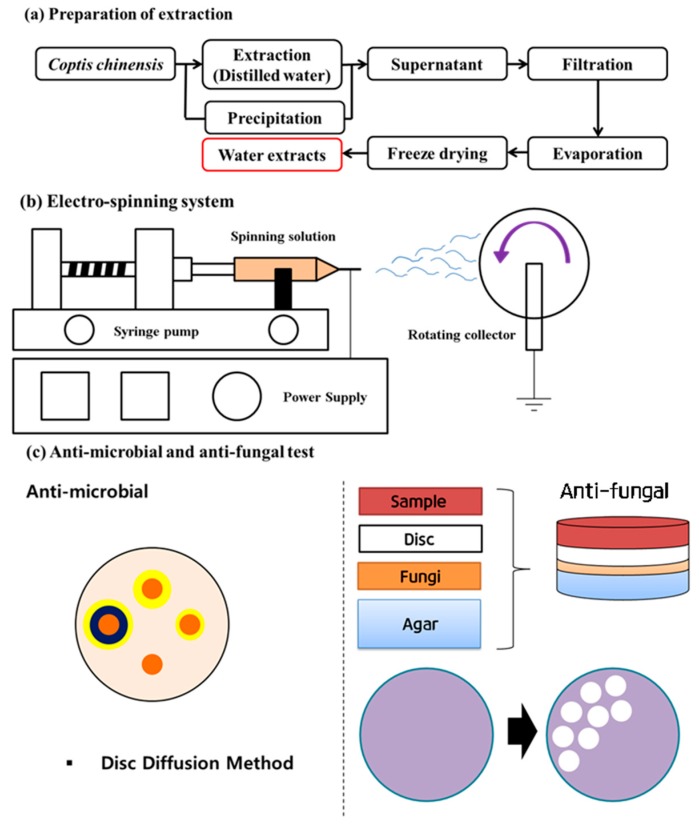
Schematic illustration of the parts of the experiment ((**a**) extraction of CC; (**b**) electrospinning of the Poly(vinyl alcohol) PVA/CC extract fibers, (**c**) anti-microbial and anti-fungal testing of the PVA/CC extract nanofibers).

Electrospinning is a commonly used technique for the electrostatic production of nanofibers, in which electric power is employed to make polymer fibers with diameters varying from 2 nm to several micrometers from polymer melts or solutions. This method is a major focus of consideration for its flexibility and ability to produce continuous fibers on the nanometer scale, which is hard to achieve using other typical technologies [10]. A schematic diagram of an electrospinning system is presented in Figure 2b.

Poly(vinyl alcohol) (PVA) is biocompatible and non-toxic. It can be produced easily and shows a high water permeability [11]. PVA has a gel-forming property when mixed with different types of solvents. These properties make PVA useful for a vast range of applications, for example, in the cosmetics, food, pharmaceutical, and packaging industries. The effect of molecular weight on electrospun fibrous PVA was discussed by Koshi et al. [12]. It is normal for differences in the molecular weight of PVA to give rise to different physical and mechanical properties [13].

Recently, researchers proved that many natural compounds can be utilized for medicinal purposes. Discovering a way to create eco-friendly, natural antimicrobial materials by combining active plant extracts has become a trend in formulating newer treatments for humans and animals. Many electrospun nanofibers made from plant extracts blended with either natural or synthetic polymers have been used for numerous biomedical purposes so far [14,15,16,17,18,19,20,21]. One such plant is *Tridax procumbentsis*, which has been successfully transferred into nanofibrous mats and blended with PVA by Ganesan et al. They also examined the antimicrobial activity of these nanofibrous mats and found that the natural potential of plants can be effectively shifted to a structure that can be used for medicinal treatments [22]. Likely, Avci et al. prepared antibacterial PVA and poly(ethylene oxide) nanofibers incorporating henna leaf extracts [23], and a wound dressing technique having an antibacterial activity was successfully prepared by PVA loaded with soursop leaf extract by Aruana and his group [24].

CC, which was the plant used in this research work, has also been studied by several other researchers to investigate its medicinal benefits and cosmetics. The anti-inflammatory influence of CC was reported in a research article [25]. In other research work, the metabolic interaction of some active constituents of CC in human liver microsomes was investigated [26]. Recently, bacterial resistance to antibiotics has become a rising problem globally, and to combat this problem, some researchers have investigated the activity of some herbs, including that of CC [27].

In this work, our aim is to combine medicinal plant resources (CC) into electrospun PVA nanofibers using PVA by the electrospinning technique, to investigate the effects of different CC extract contents (5, 10, and 15% *w*/*w*) on the morphologies, and the antimicrobial and antifungal properties of the PVA/CC extract nanofibers. Besides, we have also evaluated the effect of different molecular weights of PVA on the morphologies of the PVA/CC extract nanofibers. The prepared PVA/CC extract nanofibers were characterized by field-emission scanning electron microscopy (FE-SEM), X-ray diffraction (XRD), thermogravimetric analysis (TGA), and Fourier-transform infrared (FT-IR) spectroscopy systems. Moreover, the thermal stability of the PVA/CC extract nanofibers against *Staphylococcus aureus*, antimicrobial activity of the PVA/CC extract nanofibers against *Staphylococcus aureus* and *Staphylococcus epidermidis*, and the antifungal activity of the PVA/CC extract nanofibers against the fungi *Aureobasidium pullulans* and *Penicillium pinophilum* was also evaluated. The results of the evaluation of the antibacterial and antifungal capacities of the PVA/CC extract nanofibers suggest that they may have a beneficial application in the cosmetics and medical fields.

## 2. Materials and Methods

### 2.1. Materials

Poly(vinyl alcohol) (PVA_17_) with a number-average degree of polymerization (*P_n_*) of 1700 (fully hydrolyzed, degree of saponification = 99.9%, *M_w_* ≈ 75,000) was obtained from DC Chemical Co., Seoul, Korea. PVA_26_ was also purchased from Kuraray (Tokyo, Japan). The *P_n_* and degree of saponification of PVA_26_ (*M_w_* ≈ 110,000) were 2600 and 98–99%, respectively. Doubly distilled water was used to prepare the polymer solutions. Plant material purchased from the Korean pharmaceutical market (Daegu, Korea) was used for this research. The sliced CC was air dried and chopped. All chemicals were used without any further purification.

### 2.2. Preparation of Coptis chinensis (CC) Extract

The plants were dried in the shade for 3 days and stored at −4 °C. The extract was prepared by mixing the dried plants in water and heating this mixture at 85 °C for 3 h. After filtration, the extract was concentrated with a vacuum rotary evaporator and lyophilized for 96 h to obtain an extract powder. The whole process is presented with a schematic diagram in Figure 2a.

### 2.3. Preparation of the PVA/CC Extract Solution for Spinning and Tests

PVA was dissolved in double distilled water at 80 °C by magnetic stirring for 2 h and then cooled to room temperature for stabilization. The PVA/CC extract solutions were prepared for electrospinning, and the antimicrobial and antifungal tests. In the previous studies, the range of optimum concentration of PVA_17_ and PVA_26_ were 7.5–10% *w*/*w* and 10–12.5% *w*/*w* respectively to prepare uniform nanofibers [28,29,30]. In this study, the PVA solution was prepared by dissolving PVA at 10% *w*/*w* in water. Afterwards, various amounts (5, 10, and 15% *w*/*w* of polymer weights) of CC extracts were added separately to the PVA solutions at 25 °C with continuous stirring for another 2 h. Each of the PVA and CC extract aqueous solutions prepared were mixed for 2 h.

### 2.4. Preparation of the PVA/CC Extract Nanofibers by Electrospinning

Nanofibers were prepared by electrospinning of the pure PVA and PVA/CC extract solutions. During electrospinning, a voltage of 15 kV (Chungpa EMT Co. Ltd., Seoul, Korea; model CPS-60K02VIT) was applied to the PVA/CC solution contained in a syringe via an alligator clip attached to the syringe needle. The solution was delivered to a blunt needle tip via a syringe pump to control the flow rate of the solution. The prepared nanofibers were collected on an electrically-grounded piece of Al foil placed at a vertical distance of 150 mm from the needle tip. The needle size (length = 20 mm, diameter = 1 mm) was controlled on the spinneret for electrospinning. The electrospinning was performed at conditions of 25 °C and 60% relative humidity, and the flow rate was 0.04 mL/h.

### 2.5. Characterization of the PVA/CC Extract Nanofibers

The viscosity of the PVA and PVA/CC extract solutions was measured using a viscometer (A&D Ltd., Tokyo, Japan, SV-10) at 25 °C. Viscometer determines viscosity by identifying the driving electric current essential to vibrate the two sensor plates at 30 Hz constant frequency and amplitude of bellow 1mm. The surface morphology of the PVA and PVA/CC extract nanofiber mats was studied using a field-emission scanning electron microscope (FE-SEM, SU8220, Hitachi, Tokyo, Japan). An FT-IR spectrometer (Frontier, Perkin Elmer, Waltham, MA, USA) was utilized to analyze the CC extract and PVA/CC extract nanofibers. A reflection-type X-ray diffractometer (Philips model X-Pert APD) using a Cu Kα radiation with a wavelength of 0.154 nm was used to investigate the crystallinity of the CC extract and PVA/CC extract nanofibers. The thermal stability of the PVA/CC extract nanofibers was studied using TGA (TA Instruments, New Castle, DE, USA, Q-50) at a heating rate of 10 °C/min, from room temperature to 600 °C. Fiber diameters were evaluated from the obtained FE-SEM images using the Photoshop CS7 software (Adobe Systems Korea Ltd., Seoul, Korea). The analysis examined at least 30 different fibers and 100 different randomly selected segments from each image. The surface tension of the PVA solution and PVA/CC extract solution were measured using a TD 1C auto surface tension-meter (LAUDA, LTD, Pfarrstraße, Germany).

### 2.6. Evaluating the Antimicrobial Activity by the Disc Diffusion Method

The microorganisms used for testing the antimicrobial activity were purchased from the Korean Collection for Type Cultures (KCTC, Daejeon, Korea). *Staphylococcus aureus* (KCTC 1621) and *Staphylococcus epidermidis* (KCTC 1917) were grown at 37 °C for 24 h in trypticase soy medium and nutrient medium, respectively. The pellet was finally suspended in sterile peptone water to yield a cell density of >1 × 10^6^ CFU/mL. The paper disc diffusion method was used for measuring the antimicrobial activity. One colony of the microorganisms was selected and grown in 10 mL of each medium and incubated at 37 °C for 24 h, and then inoculated again (0.1 mL of the cell suspension) in 10 mL of the medium. Then, the inoculum (about 1 × 10^6^ CFU/mL) was aseptically spread onto the agar plates of different media. The plates were kept in a sterile workstation for 30 min to allow it to be absorbed with the designated concentration of CC extract and PVA/CC were grown at 37 °C for 24 h. The average weight of the nanofiber disk was 13 mg and the size was 8 mm in width and length. The average weight of the extract disk was 10 mg and the size was 8 mm in width and length. The antimicrobial activity was evaluated by measuring the zone of inhibition (mm) against the test microorganisms.

Most of the processed products are heat treated to efficiently control harmful organisms. Therefore, an activity of the ingredients to be added to the processed products must be maintained during the working process. Heat treatment was performed at 37, 70, 90 and 180 °C for 5 min to determine the thermal stability of PVA/CC, and then they were tested for antimicrobial activity against *Staphylococcus aureus* by the paper disc diffusion method. It can be used for processed products accompanied with heat treatment during processing.

### 2.7. Evaluating the Cytotoxicity of PVA/CC Extract Nanofiber

A cell viability assay was carried out to evaluate the cytotoxicity of pure CC extract and PVA/CC extract nanofiber containing CC extract. It was clarified by methyl thiazolyltetrazolium (MTT) assay. The CCD-986sk cells found at human fibroblast cell line were gained from American Type Culture Collection (ATCC; Manassas, VA, USA). To culture the CCD-986sk cells, Dulbecco’s modified Eagle’s medium (DMEM) 10% Fetal bovine serum (FBS) and 100 U/mL penicillin/streptomycin were used. A 96 well plate was used for seeding cells at 3 × 10^5^ cell/mL. The incubation of the cells was performed in a humidified atmosphere comprising 5% CO_2_ at 37 °C. After 24 h, cells were preserved in culture medium without FBS up to 24 h. Serum-starved cells were then treated with or without pure CC extract and PVA/CC extract for 24 h. Subsequently, the cells were incubated for 4 h with 20 μL of MTT solution in culture medium. Then the culture medium was removed with dye, added 150 μL of dimethyl sulfoxide into each well and shaken for 15 min. The absorbance was determined at a wavelength of 540 nm.

### 2.8. Evaluating the Antifungal Activity of the PVA/CC Extract Nanofibers

The modified ASTM G21 method and the modified agar diffusion method were used to determine the antifungal activity of the PVA/CC extract nanofibers. ASTM G21 antimicrobial test method is generally used to test the resistance of materials against fungal infects. Nutrient salt agar (NSA) is transferred into germfree petri dishes. A fungal plug grown on agar was placed at one end of the agar plate and a bacterial culture was streaked 2.5 cm horizontally from the fungal plug and longitudinally extended to 2 cm. The bacterial inoculum was prepared as mentioned previously. The fungal plug was placed equidistantly on the agar plate. Subsequently, a sterile filter paper disc (0.6 cm diameter) containing the PVA/CC extract nanofiber web was placed on top of each plug. A filter paper disc containing deionized water was used as a control. The plates were then incubated at 25 °C for 14 days. The fungicidal performance of the PVA/CC extract nanofibers was evaluated by using the mold index of the fungal growth area ratio on the agar surface.

## 3. Results and Discussion

### 3.1. Property of the PVA/CC Extract Solution for Electrospinning

The PVA/CC extract solution containing various CC extract contents (0, 5, 10, and 15% *w*/*w*) for electrospinning was prepared. Here, the polymer solution concentration for every solution is 10% *w*/*w*. According to previous studies, during the electrospinning process, successful spinning of nanofibers depends on some critical factors, for example, physical and chemical constraints of polymer solutions, including the viscosity, surface tension, electric conductivity, and polymer concentration [31]. Table 1 presents the viscosity, spinnability, and surface tension of the prepared PVA/CC extract solution using PVA_17_ and PVA_26_ for electrospinning, and it can be seen that the viscosities of the PVA/CC extract solution increased with increasing CC content for both the molecular weights of PVA. A careful comparison indicates that the maximum viscosity was obtained in case of PVA_26_ with 15% *w*/*w* CC content, and the opposite result was seen in case of pure PVA_17_. It is considered that in aqueous solutions the inter-and intramolecular interactions occurred between the polar hydroxyl groups of the PVA molecule due to hydrogen bonding, and consequently effect the rheological behavior of the PVA and PVA/CC extract solutions [32].

### 3.2. Morphology

**Figure 3:**
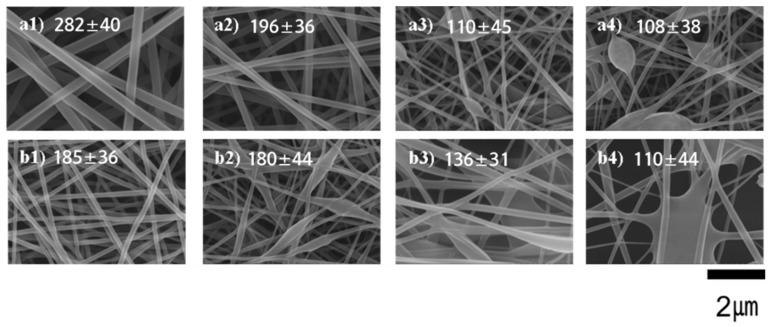
FE-SEM images of the electrospun PVA/CC extract nanofibers. All polymer concentrations are 10% *w*/*w*. (**a1**–**a4**) are samples that used PVA_26_; (**b1**–**b4**) are samples that used PVA_17_; (**a1**,**b1**) are pure PVA nanofiber samples; (**a2**,**b2**) contained 5% *w*/*w* of the CC extract; (**a3**,**b3**) contained 10% *w*/*w* of the CC extract; Finally, (**a3**,**b3**) contained 15% *w*/*w* of the CC extract. Tip to collector distance = 150 mm, Applied voltage = 15 kV.

During the electrospinning process of a polymer solution, solvent plays a key role in solution properties like conductivity, surface tension and viscosity [33]. Consequently, it is promising to make a variation in electrospun fiber morphology by modifying the solvent composition to regulate the surface tension, and solution viscosity at a fixed solution concentration. FE-SEM morphologies of electrospun PVA nanofibers with different molecular weights of PVA and various concentrations of the CC extract (0, 5, 10, and 15% *w*/*w*) are presented in Figure 3. At a fixed applied voltage (15 kV) and tip-to-collector distance (TCD) (150 mm), the typical morphology of only PVA nanofibers with different molecular weights of PVA is shown in Figure 3(a1,b1). The morphologies of the nanofibers changed progressively when the amount of CC extract was increased from 0 to 15% *w*/*w*. It can be seen that absolutely smooth, thin, and uniform fibers were easily obtained in case of both the molecular weights of PVA (Figure 3(a1,b1)). In contrast, such fibers cannot be easily obtained with PVA containing a higher concentration of the CC extract. After a sensible comparison of the FE-SEM images shown in Figure 3, we were able to determine that the preparation of bead-free fibers even with low CC extract contents using PVA_17_ is not possible (Figure 3(b2)). However, using PVA_26_ with a low concentration of CC extract (5% *w*/*w*) can produce smooth, thin, and uniform fibers, as seen in Figure 3(a2). And in case of both PVA (PVA_17_ and PVA_26_) connected fibers with beads are seen at a higher concentration of CC extract (10% *w*/*w* and 15% *w*/*w*, Figure 3(a3, a4, b3, and b4)).

### 3.3. Electrospinnability

The electrospinnability of PVA of different molecular weights containing various concentrations of the CC extract (0, 5, 10, and 15% *w*/*w*) is summarized in Table 1. From this table, it is clear that absolutely smooth, thin, and uniform nanowebs can be produced using both PVA_17_ and PVA_26_ in their pure states. However, in case of blending PVA_17_ with different concentrations of CC extract (5, 10, and 15% *w*/*w*), nanofibers cannot be produced successfully. Additionally, the fiber quality deteriorated with the increase of the CC extract concentration. On the other hand, using PVA_26_, uniform fibers can be produced using low concentrations of CC extract (5% *w*/*w*). Therefore, it is seen that PVA_17_ cannot produce uniform quality fiber with CC extract; however, this is possible by using PVA_26_ with a CC extract concentration of 5% *w*/*w*.

### 3.4. Fourier Transform Infrared Analysis

**Figure 4:**
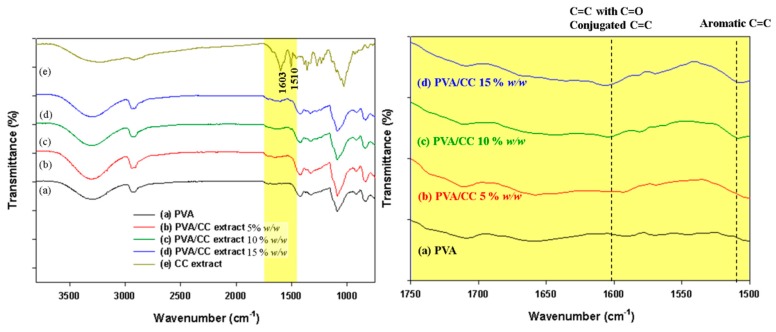
FT-IR spectra of the (**a**) electrospun PVA_26_; (**b**–**d**) electrospun PVA_26_/CC extract nanofibers; and (**e**) CC extract powder; (**b**–**d**) indicate samples containing CC extract concentrations of 5, 10, and 15% *w*/*w*, respectively). All polymer concentrations are 10% *w*/*w*. Right figure represent the magnified form of left figure.

Figure 4 shows the FTIR spectra for pure PVA_26_ and PVA_26_/CC extract nanofibers. The characteristic bands that PVA usually produce are: ν(O–H) at 3430 cm^−1^, ν(C–H_2_) at 2943 cm^−1^, and str(C=O) at 1097 cm^−1^ [34]. Compared to PVA, the major vibration bands 1510 cm^−1^ and 1603 cm^−1^, related to ν(aromatic C=C) and ν(C=C with C=O conjugated C=C), respectively, appeared in PVA_26_/CC extract nanofibers; this indicates the presence of berberin, jateorrhizine, palmatine, coptisine, magnoflorine, epiberberin, berbestine, and worenine, which are regarded as the major components of CC [2,35]. These results prove that the CC extract was successfully combined with PVA_26_.

### 3.5. XRD Data

**Figure 5:**
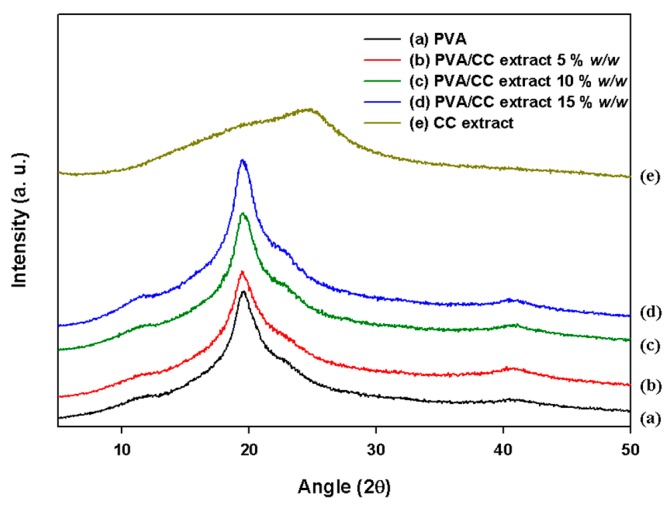
XRD analysis of the (**a**) electrospun PVA_26_; (**b**–**d**) electrospun PVA_26_/CC extract nanofibers, and (**e**) CC extract powder; (**b**–**d**) indicate samples containing CC extract concentrations of 5, 10, and 15% *w*/*w*, respectively). All polymer concentrations are 10% *w*/*w*.

The structural variations of the prepared PVA_26_/CC extract nanofibers caused by the existence of the CC extract were characterized using XRD examination. The XRD outlines obtained for the nanofibers were evaluated with those of pure PVA. It is well established that polymers comprising a crystalline region display sharp X-ray diffraction peaks with higher intensities, while amorphous polymers show broad peaks [36]. In the XRD pattern (Figure 5a), the peak located at 2θ = 19.4° is related to the (101) plane in the pristine PVA nanofibers [37]. As shown in Figure 5b–d, the location and sharpness of the corresponding hybrid peak did not change upon combination with the CC extract, this indicates that the crystallinity remained unchanged, but the chemical structure change was confirmed by FTIR (Figure 4). Moreover, there is a clear difference between the XRD patterns of pure CC and PVA_26_/CC, indicating the combination of CC extract with PVA_26_/CC.

### 3.6. Thermal Stability

**Figure 6:**
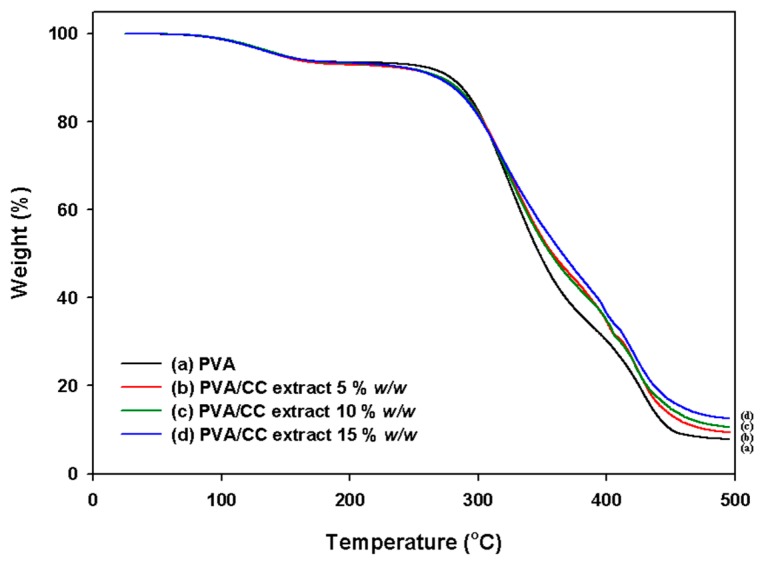
TGA curves of the (**a**) electrospun PVA_26_; (**b**–**d**) electrospun PVA_26_/CC extract nanofibers. ((**b**–**d**) indicate samples containing CC extract concentrations of 5, 10, and 15% *w*/*w*, respectively). All polymer concentrations are 10% *w*/*w.*

It is well known that during pyrolyzation, PVA undergoes dehydration and depolymerization at temperatures of over 200 and 400 °C in the absence of oxygen. The real depolymerization temperature is influenced by the molecular weight, structure, and conformation of the polymer [29]. TGA of the PVA_26_/CC extract nanofibers was performed in a nitrogen atmosphere to study their thermal stability, and the thermograms were evaluated in order to elucidate the effect of the PVA_26_/CC extract mass ratios on the thermal permanence of the nanofibers. Figure 6 shows the TGA thermograms obtained from the PVA_26_/CC extract nanofibers with different mass ratios. Three weight-loss peaks were seen in the TGA curve for pure PVA_26_ (Figure 6a). The first one, at 25–60 °C, is because of moisture vaporization, the second peak, created at 250–380 °C, is because of the thermal degradation of PVA_26_, and the last one, found at 430–480 °C, is the result of the formation of PVA_26_ byproducts throughout the thermal degradation process of the TGA. A similar tendency was observed in case of the PVA_26_/CC extract. In addition, the results suggest that thermal stability increases with the addition of CC to PVA_26_ (Figure 6b–d). More clearly, it can be established that a higher thermal stability at any stage of degradation of PVA_26_ could be obtained with higher mass ratios of the CC extract in the PVA_26_/CC extract nanofibers. These results proved that the mass ratios have a meaningful influence on the thermal properties of PVA_26_/CC extract nanofibers.

### 3.7. Antimicrobial Activity

**Figure 7:**
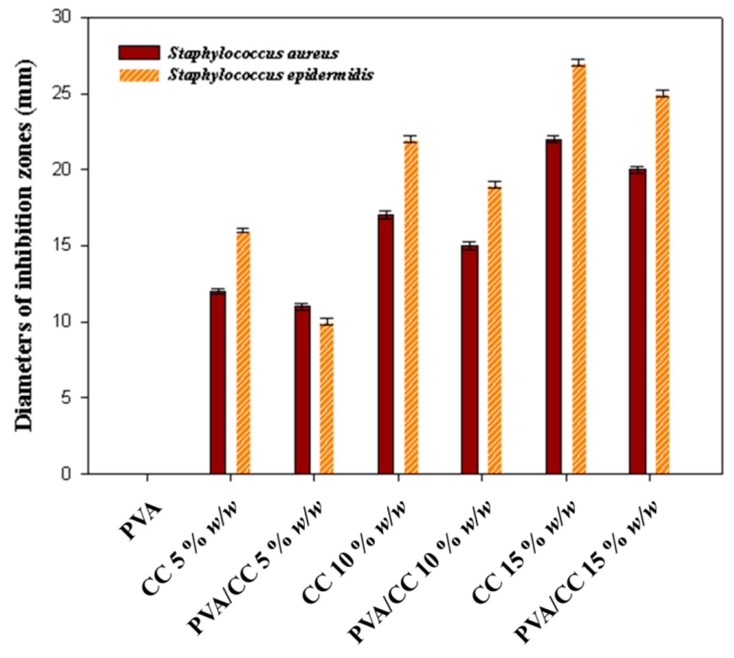
Antimicrobial activity of CC extract and PVA_26_/CC extract nanofiber against *Staphylococcus aureus* and *Staphylococcus epidermidis.* The average diameters of inhibition zones produced after treatment with CC 5% *w*/*w*, CC 10% *w*/*w*, CC 15% *w*/*w*, PVA_26_, PVA_26_/CC 5% *w*/*w*, PVA_26_/CC 10% *w*/*w*, and PVA_26_/CC 15% *w*/*w*. (A disk diameter of 10 mm was included with the diameter of the inhibition zone).

The antimicrobial activity results obtained for CC extract (5, 10 and 15% *w*/*w*) and PVA_26_/CC extract nanofibers containing different CC extract concentrations (0, 5, 10 and 15% *w*/*w*) against *Staphylococcus aureus* and *Staphylococcus epidermidis* are described in Figure 7. According to the figure, the CC and PVA_26_/CC extracts have an antibacterial activity, and inhibition zones were formed in the case of both the bacteria. It can also be observed that the CC extract and PVA_26_/CC extract shows a higher antibacterial activity against *Staphylococcus epidermidis* than *Staphylococcus aureus*; there was no distinct inhibition area (0 mm) for pure PVA_26_. The inhibition zone for pure CC 5% *w*/*w* resulted in diameter of 11.5 mm against *Staphylococcus aureus* and diameter of 16 mm against *Staphylococcus epidermidis*. And PVA_26_/CC 5% *w*/*w* created an inhibition zone (diameter of 10.1 mm) against *Staphylococcus aureus* which is not meaningfully different from that against *Staphylococcus epidermidis* (diameter of 10 mm). It can be also seen from Figure 7 that the maximum inhibition zones are caused by CC 15% *w*/*w* and PVA_26_/CC 15% *w*/*w*, respectively, in case of the growth of both the bacteria. However, PVA_26_/CC extract has slightly lower antimicrobial activity than pure CC extract. Overall the antibacterial activity of CC extract shows an almost similar trend to that of PVA_26_/CC extract. Also, these results revealed the effective antimicrobial behavior of the CC extract at higher concentrations. The observed antibacterial activity of the CC extract might be due to the presence of berberine, which is known to have an excellent antibacterial property [34].

The thermal stability test results of the PVA_26_/CC extract nanofibers against *Staphylococcus aureus* are summarized in Table 2. It showed the effect of heat treatment on the antimicrobial activity of CC extracts against *Staphylococcus aureus* at different temperatures (37, 70, 90, and 180 °C) for 24 h. The test results revealed that the PVA_26_/CC extract retained its activity even after exposure to 180 °C; there was no significant change in the antimicrobial activity when the temperature was increased from 37 to 180 °C. These findings will permit researchers to choose the suitable methods for the processing of the CC extract in order to preserve its antimicrobial capacity.

### 3.8. Cytotoxicity Effect

**Figure 8:**
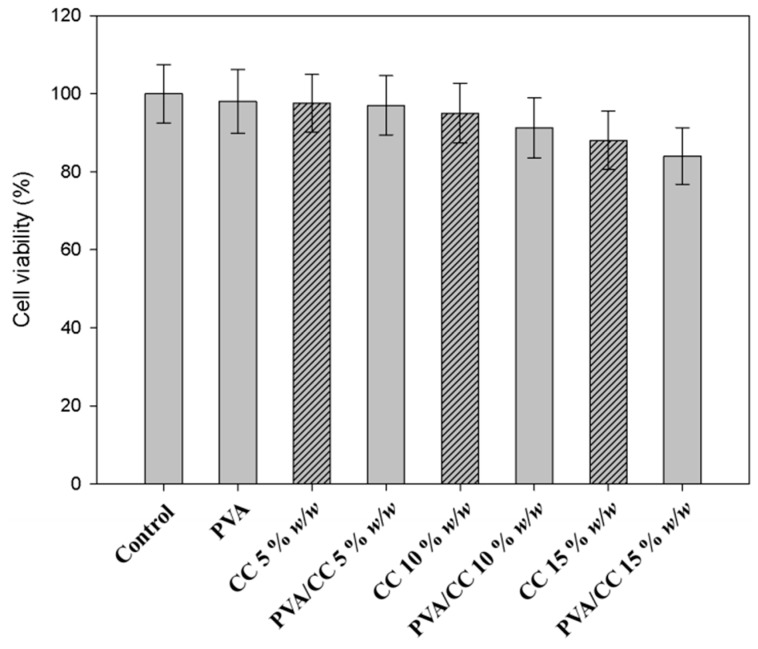
Cell viability assay of CC extract and PVA_26_/CC extract nanofiber on human skin fibroblast cells. The cells were treated with the indicated concentration of CC extract and PVA_26_/CC extract for 24 h after added the serum-free media for 24 h. And then the cell viability was examined by MTT assay. The results were expressed as the average of triplicate.

To assess the cytotoxic effect of the pure CC extract (5, 10 and 15% *w*/*w*) and PVA_26_/CC extract nanofiber on human skin fibroblasts (CCD986sk), MTT assay was executed (Figure 8). Human skin fibroblasts were incubated for 24 h with or without pure CC extract (5, 10 and 15% *w*/*w*) and PVA_26_/CC extract at various concentrations (0, 5, 10 and 15% *w*/*w*). According to test results, the cell viability surpassed 95% at a concentration of 5% *w*/*w* or less. The cell viability of 95% with the content of CC being 5% *w*/*w* or less, shows the non-toxic behavior of CC against cells.

### 3.9. Antifungal Performance

The fungicidal performance of the PVA_26_/CC extract nanofibers was evaluated by using the mold index of fungal growth area ratio on the agar surface (Table 3). Pure PVA_26_ had no antifungal activity, whereas PVA_26_/CC 5% *w*/*w*, PVA_26_/CC 10% *w*/*w*, and PVA_26_/CC 15% *w*/*w* inhibited fungal growth (Table 3). It can be also observed that the PVA_26_/CC extract nanofibers show a higher fungicidal performance against *Aureobasidium pullulans* than that against *Penicillium pinophilum*. In the presence of PVA_26_/CC 5% *w*/*w*, *Aureobasidium pullulans* resulted in a colony diameter (14 ± 0.5 mm), which was not significantly different from the colony diameter of *Penicillium pinophilum* (14 ± 0.3 mm) in the presence of PVA_26_/CC 10% *w*/*w* nanofibers. The table demonstrated the trend that the fungal growth inhibition power of PVA_26_/CC increased with increasing CC extract concentrations, and complete inhibition of the growth of both fungi is caused by PVA_26_/CC 15% *w*/*w*. These results revealed the effective antifungal behavior of the CC extract at higher concentrations. The excellent antifungal activity of CC extract might be due to its active constituent magnoflorine, which is known for its effective antifungal activity [5].

## 4. Conclusions

PVA/CC extract nanofibers containing different CC extract concentrations (0, 5, 10, and 15% *w*/*w*) were successfully prepared using the electrospinning method, and their potential use as antibacterial and antifungal agents was evaluated. Moreover, morphological changes and electrospinnability were examined using different molecular weights of PVA. The TGA results showed that the introduction of CC extract results in an improvement in the thermal stability of the PVA_26_ matrix; however, according to the XRD examination, crystallinity was unchanged after the addition of the CC extract. The existence of the major components of the CC extract was confirmed by FTIR; this confirmed the successful incorporation of the CC extract into PVA_26_. In addition, PVA_26_/CC extract nanofibers show a potential antibacterial and antifungal capacity against the bacteria *Staphylococcus aureus* and *Staphylococcus epidermidis*, and the fungi *Aureobasidium pullulans* and *Penicillium pinophilum*, respectively. Also it is found that prepared nanofibers retained its antimicrobial activity against at *Staphylococcus aureus* higher temperature. This indicates that they could be used in the medical and cosmetics fields.

## Figures and Tables

**Table 1:** The viscosity, spinnability and surface tension of the PVA and PVA/CC extract solutions using PVA_17_ and PVA_26_ for electrospinning.

Polymer	Concentration of CC Extract (% *w*/*w*)	Viscosity (mPa·s)	Spinnability	Surface Tension (mN/m)
PVA_17_	0% *w*/*w*	930	[++]	58.3 ± 3.5
	5% *w*/*w*	1565	[Δ]	57.2 ± 3.8
	10% *w*/*w*	1850	[Δ]	59.2 ± 4.8
	15% *w*/*w*	2200	[−]	60.4 ± 4.2
PVA_26_	0% *w*/*w*	1000	[++]	58.5 ± 4.2
	5% *w*/*w*	2330	[++]	61.8 ± 3.2
	10% *w*/*w*	2500	[+]	63.4 ± 4.5
	15% *w*/*w*	2600	[Δ]	64.5 ± 3.8

++: Excellent, +: common, Δ: bad, −: worst.

**Table 2:** The determination of the thermal stability of PVA_26_/CC extract (10% *w*/*w*) nanofibers against *Staphylococcus aureus.*

Temperature (°C)	Diameters of Inhibition Zone (mm)	
	Control	PVA_26_/CC 10% *w*/*w*
37	0	19 ± 0.5
70	0	18 ± 0.3
90	0	19 ± 0.4
180	0	18 ± 0.5

**Table 3:** The antifungal activity of the PVA_26_/CC extract nanofibers against *Aureobasidium pullulans* and *Penicillium pinophilum*. The average diameters of the inhibition zones produced after treatment with PVA_26_, PVA_26_/CC 5%, PVA_26_/CC 10%, and PVA_26_/CC 15% include a disk diameter of 10 mm.

Concentration of CC Extract (% *w*/*w*)	Colony Diameter (mm)	
	*Aureobasidlum pullulans*	*Penicilium pinophilum*
Blank	36 ± 0.5	34 ± 0.5
5	14 ± 0.5	20 ± 0.4
10	0	14 ± 0.3
15	0	0
